# Benzothiazole amphiphiles promote RasGRF1‐associated dendritic spine formation in human stem cell‐derived neurons

**DOI:** 10.1002/2211-5463.12788

**Published:** 2020-01-30

**Authors:** Jessica L. Cifelli, Kyle R. Berg, Jerry Yang

**Affiliations:** ^1^ Department of Chemistry and Biochemistry UC San Diego La Jolla CA USA

**Keywords:** benzothiazole, dendritic spine, neural stem cells, spinogenesis

## Abstract

Synaptic dysfunction has been implicated as an early cause of cognitive decline in neurodegenerative diseases (NDDs) such as Alzheimer’s disease (AD). Methods to slow down or reverse the loss of functional synapses, therefore, represent a promising avenue to explore for treating NDDs. We have previously reported the development of a class of benzothiazole amphiphiles (BAMs) that exhibited the capability to improve memory and learning both in wild‐type mice and in an AD rodent model, putatively through promoting RasGRF1‐associated formation of dendritic spines in hippocampal neurons. While these results represent a good first step in exploring a new approach to treating NDDs, the capability of these compounds to increase spine density has not been previously examined in a human neuronal model. Here, we found that neurons derived from differentiated human induced pluripotent stem cells exhibited both an increase in RasGRF1 expression and a phenotypic increase in the density of postsynaptic density protein 95‐positive puncta (which we use to provide an estimate of dendritic spine density) in BAM‐treated vs. control neurons. These results demonstrate that the previously observed spinogenic effects of BAMs in rodent neurons can be recapitulated in a human neuronal model, which further supports the potential utility of BAM agents for treating human diseases associated with spine deficits such as AD or other NDDs.

## Graphical abstract

Methods to promote dendritic spine formation or prevent dendritic spines loss represent a novel approach to combat memory deficits in neurodegenerative diseases. Using induced pluripotent stem cell‐derived neurons as the first human cell model capable of evaluating dendritic spine dynamics, we show that benzothiazole amphiphiles can activate cell machinery in human neurons to promote the formation of dendritic spines.

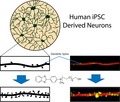

**Abbreviations**
AD: Alzheimer’s disease
BAM: benzothiazole amphiphile
BDNF: brain‐derived neurotrophic factor
dbcAMP: dibutyryl cyclic adenosine monophosphate
FGF: fibroblast growth factor
iPSC: induced pluripotent stem cell
NDDs: neurodegenerative diseases
NSC: neuronal stem cell

Synaptic dysfunction is a hallmark feature in many neurodegenerative disorders. Synapse loss was first correlated to Alzheimer’s disease (AD) in 1987 1 and was later suggested to be a major link to memory loss in AD 2. Additionally, synapse dysfunction and loss have been implicated in many other forms of dementia including spongiform encephalopathy 3, vascular dementia 4, and frontal lobe degeneration 5. However, there are no current approved methods to slow or halt the progressive synaptic or neuronal loss in neurodegenerative diseases (NDDs) 6. The functional unit of the synapse consists of both presynaptic and postsynaptic structures 7, which contain the necessary machinery to transmit neurochemical signals across the synaptic cleft 8. The postsynaptic structure, known as the dendritic spine, is actively remodeled and recycled on the surface of the dendrite 9, 10, 11, and the density of dendritic spines in the hippocampus is positively associated with memory and learning 12, 13. Methods to increase the density of dendritic spines, therefore, may represent a novel approach to slow down or counteract the cognitive effects of synapse loss in NDDs.

To date, relatively few compounds have been shown to increase dendritic spine density 14, 15, 16, 17, 18, 19, 20, 21, 22. For instance, we have previously reported that benzothiazole amphiphiles (BAMs) can increase dendritic spine density in primary rat hippocampal neuronal culture 15 as well as *in vivo* in wild‐type mice and in a 3xTg mouse model for AD 23, 24, 25. These initial reports showed that BAMs are able to improve memory and learning in rodents by a RasGRF1 (a guanine nucleotide release factor involved in regulation of dendritic spine formation)‐associated mechanism for promoting dendritic spine formation. Here, we examine whether the effects of BAM compounds on dendritic spine density found in rodent neurons can also be replicated in human neuronal models. Toward this end, we choose three different human neuronal model systems to evaluate efficacy of three different BAM compounds for their capability to promote RasGRF1‐dependent increases in dendritic spine density: undifferentiated SH‐SY5Y neuroblastoma cells, retinoic acid (RA)‐induced differentiated SH‐SY5Y neuroblastoma cells, and mature neurons derived from human‐induced pluripotent stem cells (iPSCs). We estimated dendritic spine formation by measuring postsynaptic density protein 95 (PSD95)‐positive puncta, a protein that is commonly found in high levels in mature dendritic spines. We found that neurons derived from iPSCs were the best human model system for evaluating spinogenic activity of BAM compounds, as these neurons treated with BAMs **1‐3** (Fig. 1) displayed a significant increase in density of PSD95 puncta compared to vehicle treatment. Additionally, RasGRF1 expression was found to be increased in the BAM‐treated neurons compared to the control neurons. Together, these results indicate that the same machinery found activated in rat primary culture by BAMs can also be activated in human neurons. To our knowledge, this report represents the first example of the use of human iPSC‐derived neurons to examine the biochemical and phenotypic activity of promoters of dendritic spine density, which could be useful for developing such compounds for treating human diseases.

**Figure 1:**
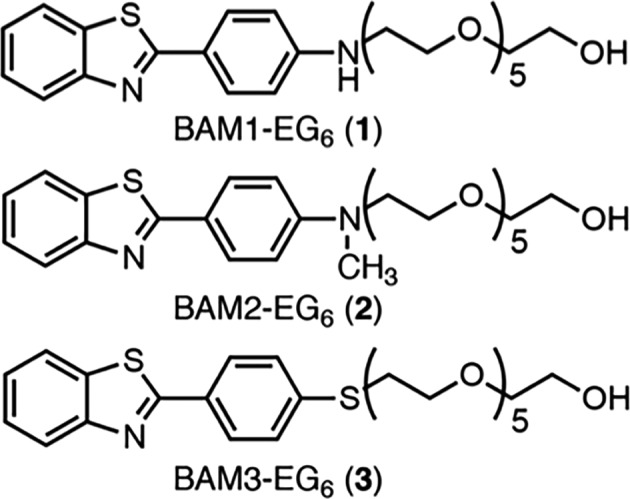
Structures of BAMs **1‐3**.

## Materials and methods

### Materials

Human iPSC‐derived neuronal stem cells (NSCs) were a generous gift from L. Goldstein (Department of Cellular and Molecular Medicine, UCSD). Dulbecco’s modified Eagle’s medium (DMEM)/F12 + Glutamax (#10565‐018), N‐2 supplement (#17502‐048), and B27 supplement (#170504‐044) were purchased from Gibco (Gaithersburg, MD, USA). Human basic fibroblast growth factor (bFGF) (#100‐18B) and Brain‐derived neurotrophic factor (BDNF) (#450‐02B) were purchased from PeproTech (Rocky Hill, NJ, USA). Penicillin–streptomycin (Pen/Strep) (#17‐602E) was purchased from Lonza (Basel, Switzerland). Accutase (AT‐104) was purchased from Innovative Cell Technologies, Inc. (San Diego, CA, USA). Sterile 0.4 μm cell strainers (352340) were purchased from BD Falcon (Franklin Lakes, NJ, USA). Laminin (#L2020) and poly‐l‐ornithine (#P3655) were purchased from Sigma (St. Louis, MO, USA). Coverslips, 25 mm #1 German Glass (#CLS‐1760‐025), were purchased from Chemglass (Vineland, NJ, USA). Six‐well culture plates (#353046) were purchased from Falcon. Human Neural Stem Cell Immunocytochemistry Kit (#A24354) was purchased from Molecular Probes by Life Technologies (Carlsbad, CA, USA). Primary antibodies used were as follows: mouse anti‐Nestin (Life Technologies #A24345), rabbit anti‐SOX2 (Life Technologies #A24339), mouse anti‐MAP2 (Sigma #M1406), rabbit anti‐PSD95 (Invitrogen #51‐6900, Carlsbad, CA, USA), mouse anti‐RasGRF1 (BD 610149), and mouse anti‐GADPH (Sigma G8795). Secondary antibodies used were as follows: Alexa Fluor® 488 donkey anti‐mouse (Life Technologies #A24350), Alexa Fluor® 555 donkey anti‐rabbit (Life Technologies #A24342), TRITC‐conjugated goat anti‐mouse (Jackson ImmunoResearch, West Grove, PA, USA), Alexa Fluor® 488‐conjugated goat anti‐rabbit (Jackson ImmunoResearch), and ECL™ Horseradish Peroxidase (HRP) linked anti‐mouse (GE #NA931, Issaquah, WA, USA). Amersham™ ECL™ Prime western blotting Detection Reagent (RPN2232) was purchased from GE Healthcare. Protease inhibitor tablets (# 05892791001) were purchased from Roche (Basel, Switzerland). Pierce™ bicinchoninic acid Assay Kit (#23225) was purchased from Thermo Scientific (San Diego, CA, USA).

### SH‐SY5Y neuroblastoma cultures

SH‐SY5Y human neuroblastoma cells were grown in 1 : 1 Eagle’s minimum essential medium (EMEM) and Ham’s F12 supplemented with 10% FBS. Cells were maintained in a humidified incubator at 37 °C in 95% air and 5% carbon dioxide (CO_2_). Complete media were exchanged two times per week.

### Differentiated SH‐SY5Y neuroblastoma cultures

SH‐SY5Y neuroblastoma cells were differentiated as previously described 26. Briefly, SH‐SY5Y human neuroblastoma cells were grown in complete medium (1 : 1 EMEM and Ham’s F12 supplemented with 10% FBS). After adhering overnight, cells were differentiated by the addition of 10 μm all‐*trans*‐RA to the complete medium. Medium with RA was replaced every 2 days for 8 days total. Cells were maintained in a humidified incubator at 37 °C in 95% air and 5% CO_2_. Differentiation of SH‐SY5Y cells by RA was monitored over the course of 9 days, and we observed large changes in cell morphology accompanied by substantial neuritic outgrowth as has been reported previously for this cell line 27.

### Human iPSC‐derived neural stem cell culture

Human iPSC‐derived, FACS‐purified, NSCs were maintained in base medium composed of DMEM/F12 medium with Glutamax supplemented with N‐2 (0.5×), B‐27 (0.5×), bFGF (20 ng·mL^−1^), and Pen/Strep (1×). bFGF was added fresh every week. Cells were plated on 6‐well plates that were coated with laminin (5 μg·mL^−1^) and poly‐l‐ornithine (20 μg·mL^−1^) and incubated at 37 °C, 5% CO_2_, and 95% humidity. Base medium was changed every 2–3 days, and cells were split at a ratio of 1 : 3 at 80–90% confluency with Accutase.

### Human iIPSC‐derived neural stem cell differentiation

For the differentiation of human iPSC‐derived NSCs into mature neurons, base medium was used [DMEM/F12 medium with Glutamax supplemented with N‐2 (0.5×), B‐27 (0.5×), bFGF (20 ng·mL^−1^), and Pen/Strep (1×)] with the addition of BDNF (20 ng·mL^−1^) and dibutyryl cyclic adenosine monophosphate (dbcAMP; 0.5 mm) (here, no bFGF was added). This differentiation medium was changed every 3–4 days, and cells were maintained at 37 °C, 5% CO_2_, and 95% humidity. Cells were plated on either 6‐well dishes, MatTeks, or 8‐well chamber slides coated with laminin (5 μg·mL^−1^) and poly‐l‐ornithine (20 μg·mL^−1^) and let differentiate for up to 3 months.

### Immunocytochemistry of human iPSC‐derived neural stem cells

A Human Neural Stem Cell Immunocytochemistry Kit (A24354) was utilized for the characterization of human NSCs. The kit was used to confirm expression of the common undifferentiated precursor markers Nestin as well as SOX2. Briefly, medium was removed and cells were rinsed with PBS. Cells were fixed with the provided fixative solution [4% PFA in Dulbecco’s phosphate‐buffered saline (DPBS)] for 15 min at room temperature (RT), followed by permeabilization with the provided permeabilization solution (0.5% Triton X‐100 in DPBS) for 15 min at RT. After permeabilization, cells were blocked with the blocking solution (3% BSA/DPBS) for 1 h at RT. After removal of blocking solution, primary antibodies diluted to 1X in blocking solution were added and let incubate at 4 °C overnight. After washing (3×), the appropriate secondary antibodies were added (diluted to 1X in blocking solution) and incubated for 1 h at RT. Cells were washed 3× with two drops per mL of the provided NucBlue® Fixed Cell Stain (DAPI) added in the last wash step and allowed to incubate for 5 min. Slides or coverslips were mounted with Vectashield® and imaged with an FV1000 confocal microscope (Olympus, Waltham, MA, USA).

### Immunocytochemistry for PSD95/MAP2 staining

Cells were stained for microtubule‐associated protein 2 (MAP2), a neuron‐specific marker, as well as PSD95, a postsynaptic marker. Briefly, cells were dosed for 24 h with or without BAMs **1‐3** (5 μm in PBS). After dosing, cells were rinsed (3× PBS), fixed with 4% PFA (15 min, RT), permeabilized with 0.3% Triton X‐100/DPBS (10 min RT), blocked with 3% BSA/DPBS (1 h, RT), and incubated overnight at 4 °C with primaries: MAP2 (1 : 500) and PSD95(1 : 1000) in blocking solution. Secondaries were diluted 1 : 250 in blocking solution and let incubate for 1 h at RT. Slides or coverslips were mounted with Vectashield® and imaged with an FV1000 confocal microscope (Olympus). PSD95‐positive puncta were quantified per micron along MAP2‐positive neurons. For each treatment, three separate cultures were used and, among those cultures, a minimum of 9 total neurons were evaluated for counting the density of PSD95‐positive puncta.

### Western blot quantification of RasGRF1 expression

We performed western blot analyses to quantify protein expression of either dosed or control cells. Briefly, cells were dosed for 24 h with or without BAMs **1‐3** (0 or 5 μm in PBS). After dosing, cells were rinsed on ice (3× with ice‐cold PBS), lysed with RIPA buffer with protease inhibitors. Lysates were agitated for 30 min at 4 °C, followed by centrifugation at 4 °C (20 min, 13 000 ***g***). Supernatants were aspirated into a new ice‐cold tube, and a bicinchoninic acid assay was performed to determined protein concentration. Lysates were frozen and stored at −80 °C until use for western blot analysis. Briefly, proteins were separated by SDS/PAGE followed by transfer onto nitrocellulose membranes. Membranes were blocked with either 5% BSA or milk in Tris‐buffered saline with Tween‐20, followed by incubation with primary antibodies overnight at 4 **°**C with shaking. Proteins were visualized using the appropriate HRP‐labeled secondary by ECL, and detection was carried out on film (Freedom Imaging, SRX‐101A). The density of each band was then quantified using imagej (National Institutes of Health, Bethesda, MD, USA) software.

## Results and discussion

### BAMs marginally increase RasGRF1 levels but do not promote the expression of PSD95 in undifferentiated SH‐SY5Y neuroblastoma cells

**Figure 2:**
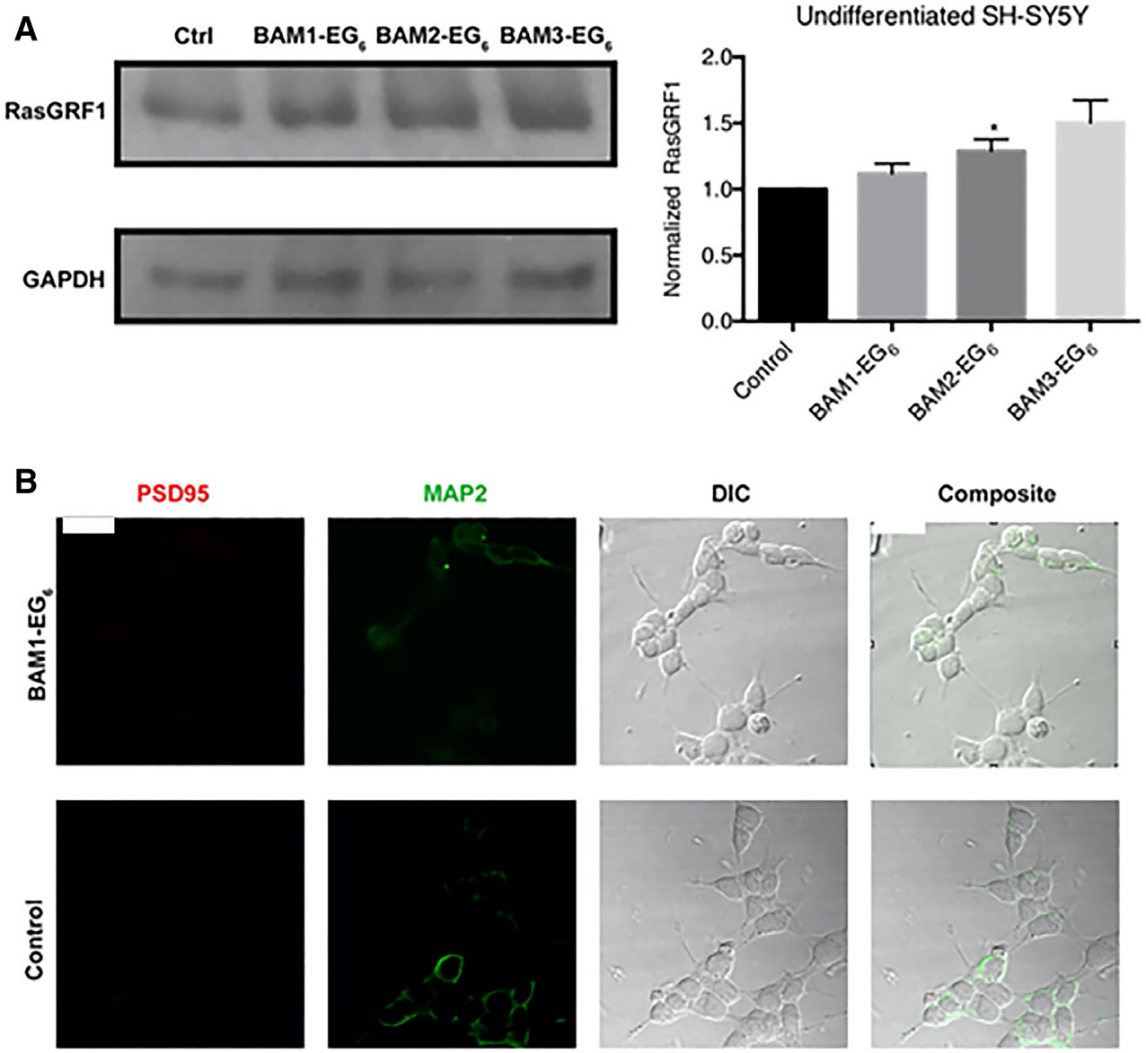
Evaluation of BAM efficacy in SH‐SY5Y neuroblastoma. (A) Comparison of relative expression levels of RasGRF1 between SH‐SY5Y cells dosed with vehicle control (PBS) or BAMs **1‐3** (5 µm). (B) Representative images of cells stained for PSD95 (red) and MAP2 (green). Data are expressed as mean values ± SEM, *n* ≥ 3, *P < 0.05 as determined by unpaired *t*‐test compared to control. Scale bar = 20 microns.

In order to initially examine whether the BAM compounds activated the same RasGRF1‐dependent spinogenic pathway in human cells as observed in rat hippocampal neurons, we attempted to utilize the neuroblastoma cell line, SH‐SY5Y, as the simplest of the three human cell model systems tested 28. The SH‐SY5Y neuroblastoma cell line was specifically chosen because of its human origin and because these cells have been reported to exhibit neuron‐like properties, especially upon differentiation 27, 28. We dosed SH‐SY5Y cells with 5 μm concentrations of BAMs **1‐3** or PBS (as vehicle control) for 24 h, followed by lysis and probing by western blot to analyze RasGRF1 expression levels. RasGRF1 was previously found to have the highest change in expression levels in a RasGRF1/Ras/ERK‐dependent pathway for increasing dendritic spines in response to exposure of the BAM compounds in rodent models 14, 15. As such, we sought to use changes in RasGRF1 expression for evaluating the potential activation of the spinogenesis pathway by these compounds. We observed only a slight increase in RasGRF1 expression for cells treated with any of the three BAM compounds, with only BAM2‐EG_6_ producing a statistically significant increase (Fig. 2A). These results are in contrast to the significant increase in Ras‐GRF1 expression levels found in primary rat hippocampal neurons dosed with the same concentrations of BAMs **1‐3** 14. These opposing results could be due to lower abundance of neuronal and synaptic proteins found in undifferentiated neuroblastoma cells compared to mature mammalian neurons 29. This low expression of synaptic proteins in SH‐SY5Y cells was further supported by immunocytochemistry experiments. Dendritic spines (the major site of excitatory synaptic input) appear on mature neurons and comprise a high local concentration of the PSD95 30. Microtubule‐associated protein 2 is typically used as a biomarker to locate dendritic arborization in neurons 31. While concurrent visualization of PSD95 and MAP2 has been used previously to identify spines in neurons, 32 high levels of PSD95 are not a requirement for all mature spines 33. Measuring density of observed PSD95 puncta may, therefore, only serve as an estimate of the density of dendritic spines in these cell studies. The SH‐SY5Y neuroblastoma cells showed undetectable levels of PSD95 (Fig. 2B), with or without exposure of cells to BAM1‐EG_6_ (as a representative spine‐promoting agent). The data indicate that undifferentiated SH‐SY5Y cells do not function as an adequate model for the evaluation of dendritic spine density.

### BAMs increase RasGRF1 levels but do not promote the formation of PSD95 puncta in differentiated SH‐SY5Y neuroblastoma cells

Since the BAM compounds are known to affect synapse formation, we next turned to differentiated SH‐SY5Y cells. SH‐SY5Y neuroblastoma cells have been shown to differentiate into a more neuron‐like phenotype through the use of RA treatment to increase neuritic outgrowth 27. We observed large morphological changes in SH‐SY5Y cell cultures over 9 days of differentiation with RA. In addition, differentiation of these cells has been reported to increase dendritic arborization and the expression of synaptic proteins 28. The expression of these synaptic proteins is most likely vital for the capability of the BAM agents to promote dendritic spine formation.

**Figure 3:**
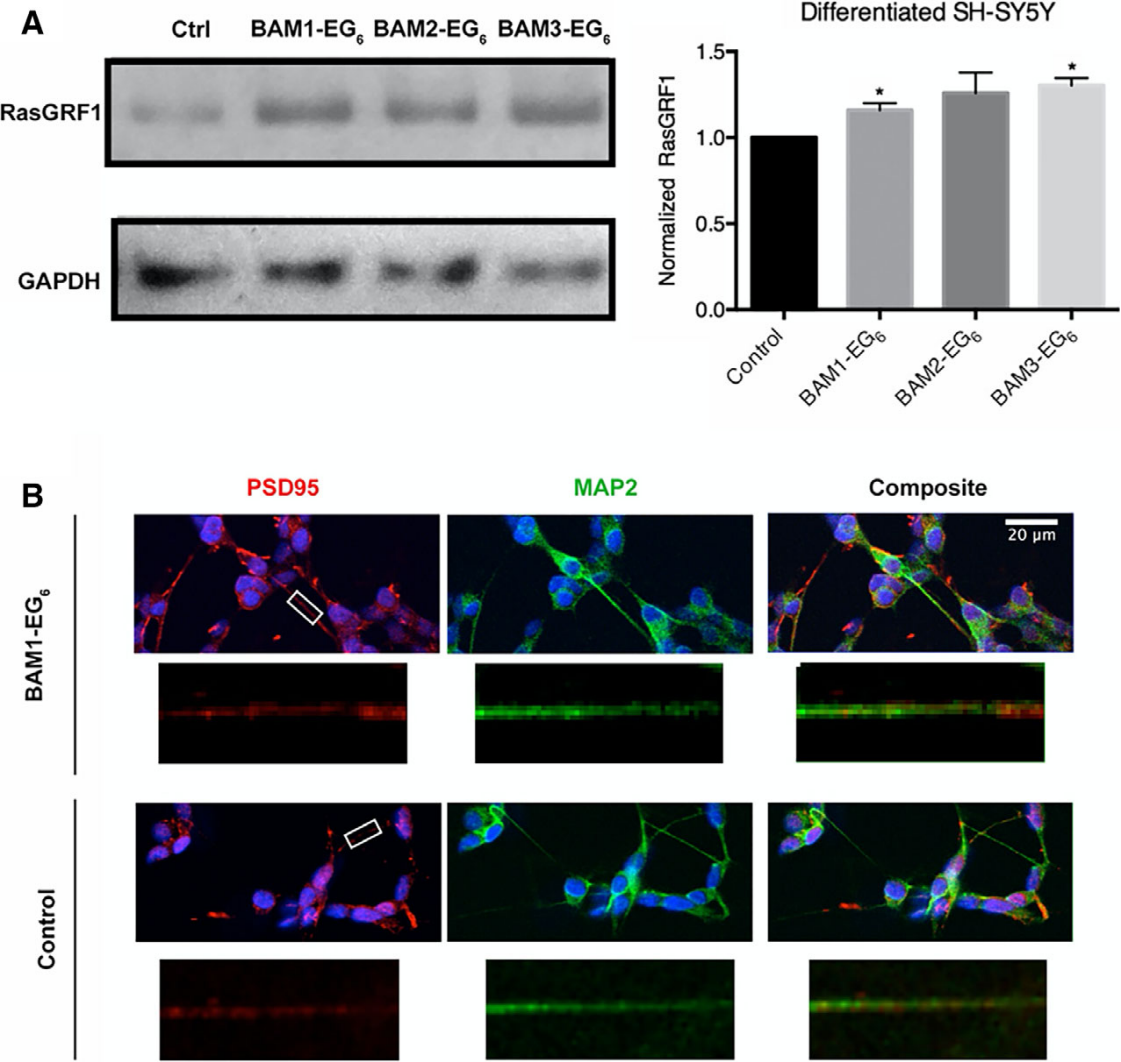
Evaluation of BAM efficacy in differentiated SH‐SY5Y neuroblastoma. (A) Comparison of relative expression levels of RasGRF1 between differentiated SH‐SY5Y cells dosed with vehicle control (PBS) or BAMs **1‐3** (5 µm). (B) Representative images of cells stained for PSD95 (red) and MAP2 (green). White boxes denote areas of segments shown in higher magnification below either BAM1‐EG_6_ dosed or control cells, respectively. Data are expressed as mean values ± SEM, *n* ≥ 3, *P < 0.05 as determined by unpaired *t*‐test compared to control. Scale bar = 20 microns.

Differentiated SH‐SY5Y cells were dosed with BAM compounds, lysed, and probed by western blot to analyze for changes in RasGRF1 expression levels compared to control cells. Here, we observed a more consistent increase in expression of RasGRF1 for cells dosed with BAM compounds compared to cells exposed only to PBS vehicle control (Fig. 3A). The observed ~ 20% increase in expression of RasGRF1 levels in the presence of BAM agents was similar to the increase in RasGRF1 levels observed in rodent models exposed to these compounds 14. For this experiment, we attempted to quantify the number of PSD95‐positive puncta along MAP2‐labeled neurites in treated vs. control cells. While we found intracellular areas with increased PSD95, we did not find any overt puncta containing PSD95 that would be consistent with a dendritic spine in the presence or absence of the BAM agents (Fig. 3B). These results highlight a potential drawback for using neuroblastoma cells for analysis of PSD95‐positive puncta, even when differentiated with RA, as they do not appear to exhibit adequate neuron‐like properties for analysis of the memory‐enhancing phenotypic activity of BAM compounds.

### Differentiation of human iPSC‐derived neural stem cells (NSCs)

Induced pluripotent stem cells represent exciting and potentially more reliable models than immortalized cells for evaluating the activity of potential drug candidates for human diseases 34, 35. Several attractive attributes of using human iPSC technology for evaluating efficacy of bioactive compounds include the following: (a) the ability to provide a continuous supply of normal (i.e., nonimmortalized) human cells 36, (b) the capability to differentiate pluripotent cells to generate specific cell types 37, 38, and (c) the unique capacity to generate either disease or healthy patient‐derived cells 39, 40, 41. Importantly for the work presented here, iPSCs have been shown to successfully differentiate to neural stem cells (NSCs) and subsequently into mature neurons 42, 43.

**Figure 4:**
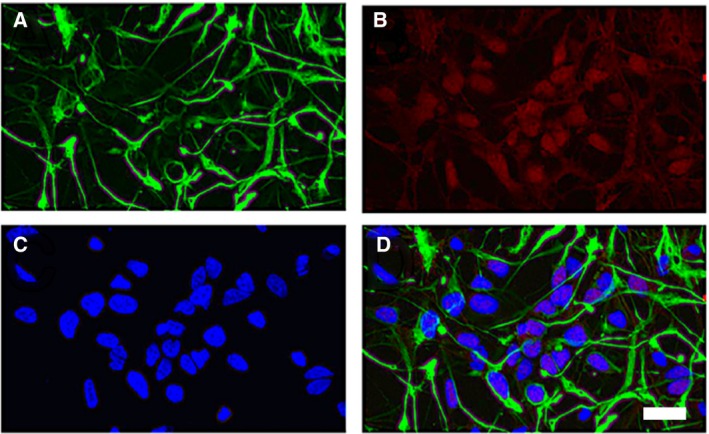
Immunohistochemistry characterization of NSCs. Undifferentiated precursor‐specific marker staining of (A) Nestin and (B) SOX2. Additional images of the (C) staining of nuclei with DAPI and (D) composite image of A‐C. Scale bar = 25 μm.

We derived human NSCs from human iPSCs as previously described by Carson and coworkers 44. The isolated NSCs were initially characterized by the undifferentiated precursor markers Nestin (an intermediate filament protein) and SOX2 (a transcription factor essential for pluripotency) (Fig. 4). These results indicate that the iPSCs were successfully differentiated into NSCs 44, 45.

**Figure 5:**
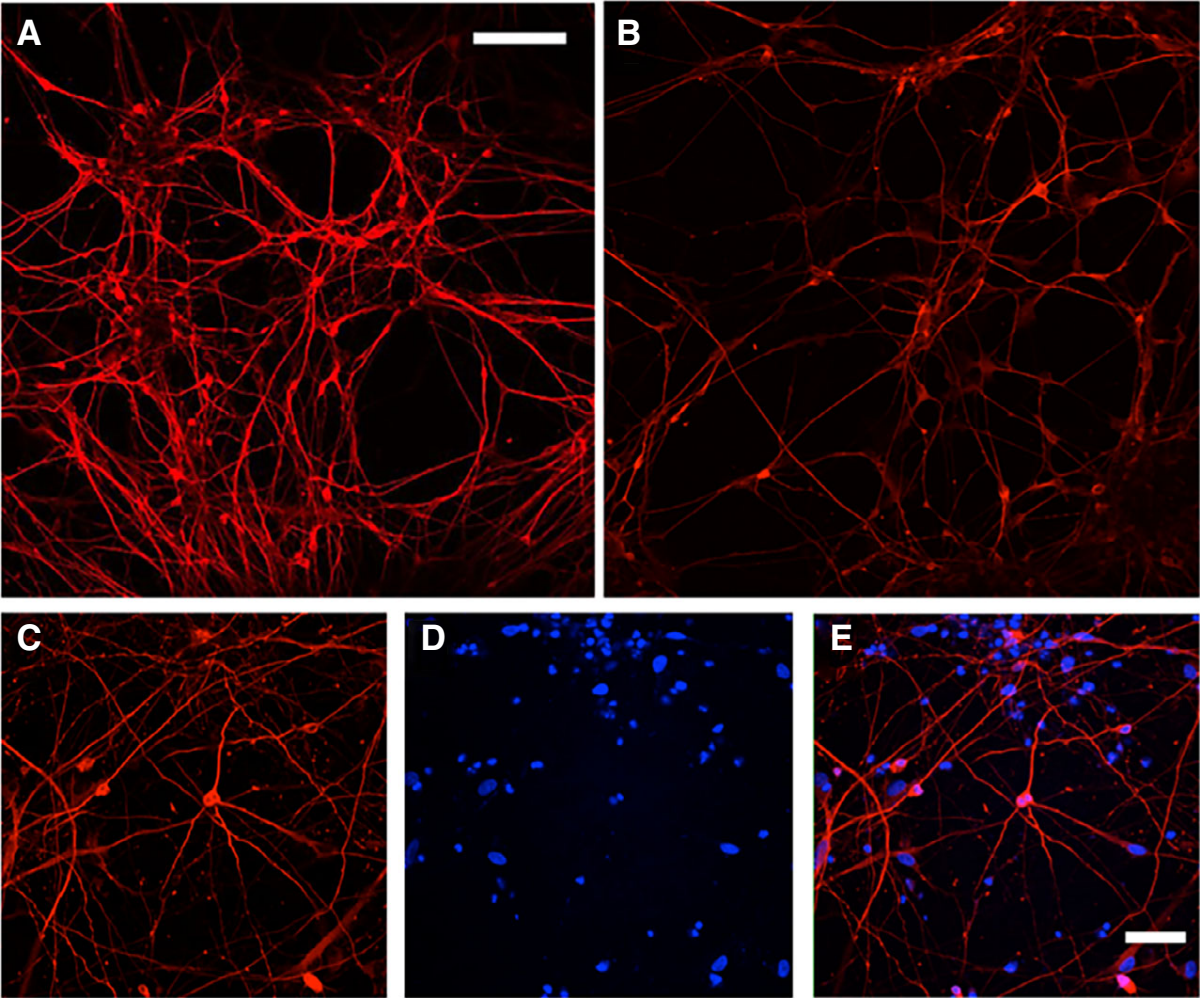
Immunohistochemistry of the neuronal‐specific marker MAP2 from three different representative preparations of human neurons derived from NSCs. MAP2 staining from preparations 1 (A), 2 (B), and 3 (C–E). DAPI staining (D) and composite (E) of images C and D. Top panel scale bar = 100 μm, bottom panel scale bar = 50 μm.

After characterization, the NSCs were differentiated into mature neurons using a previously described method of FGF removal and addition of BDNF and dbcAMP 40, 41. The differentiation process was monitored weekly by live‐cell microscopy and maintained until ready for use. Additionally, mature neurons from several different preparations were then stained with the neuronal‐specific marker, MAP2 (images of cells from three such preparations are shown in Fig. 5).

### BAMs increase RasGRF1 levels and increase the density of PSD95 puncta in human iPSC‐derived mature neurons

**Figure 6:**
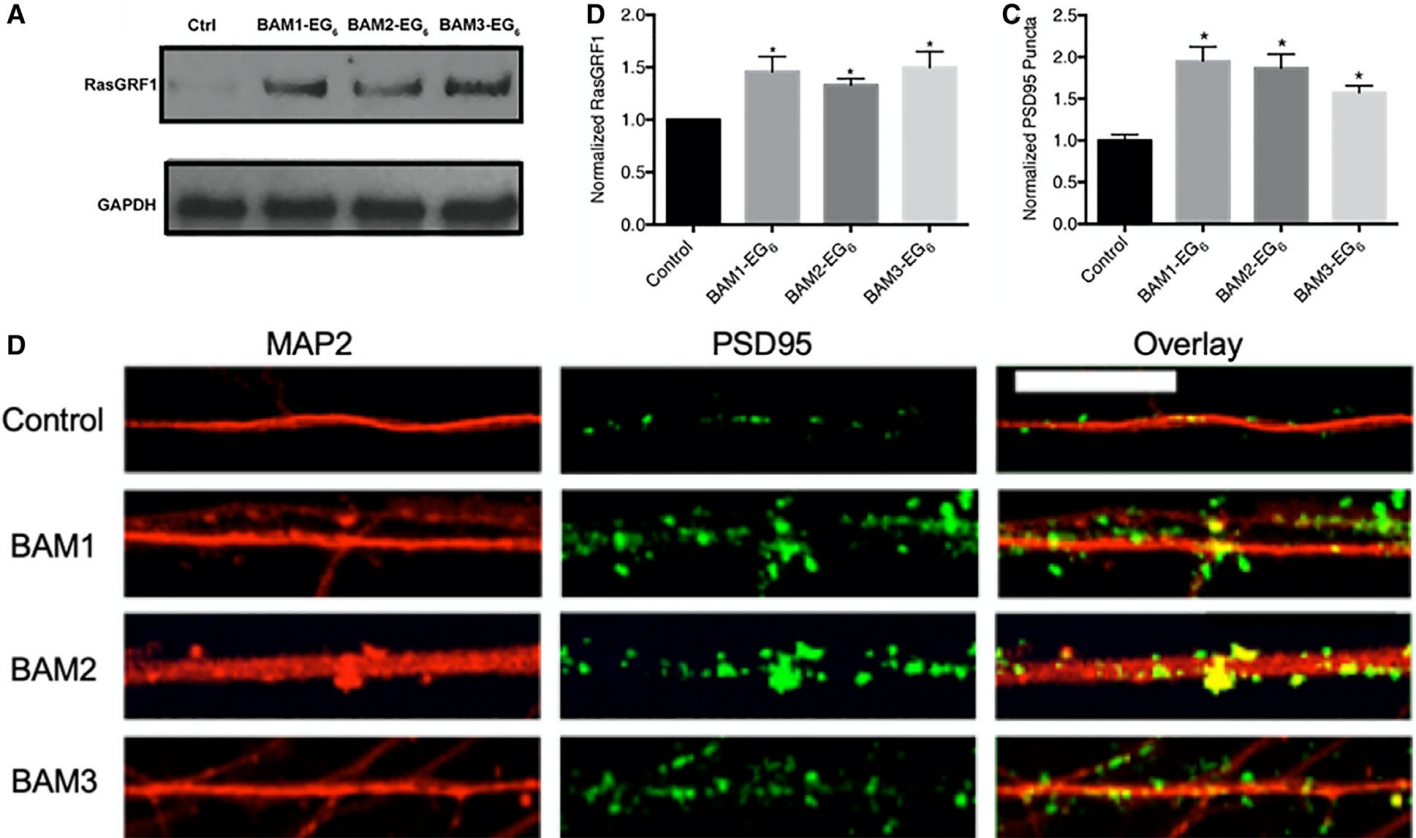
Evaluation of the activity of BAM **1‐3** in neurons derived from iPSCs. (A) Blot comparing the expression of RasGRF1 and GAPDH. (B) Comparison of relative expression levels of RasGRF1 between neurons dosed with vehicle control (PBS) or BAMs **1‐3** (5 µm). (C) Quantitative representation of the relative density of PSD95 puncta along MAP2‐positive dendrites on iPSC‐derived neurons dosed with vehicle control or BAMs **1‐3**; for clarity, the data for density of PSD95 puncta are normalized to the density on the control neurons. Data are expressed as mean values ± SEM, *n* ≥ 9 neurons selected among three separate cultures, *P < 0.05 as determined by unpaired *t*‐test compared to control. Scale bar = 25 microns. (D) Representative images of dendrite segments.

After differentiating human NSCs into mature neurons, we next sought to examine whether the BAM agents induced increased expression of RasGRF1 in these cells. For this experiment, 3‐month‐old mature iPSC‐derived neurons were exposed to solutions of BAMs **1‐3** in PBS (0 or 5 μm) for 24 h. Subsequently, cells were lysed and western blot analysis was performed in order to quantify any changes in RasGRF1 expression. We observed a significant increase in RasGRF1 expression in iPSC‐derived neurons that were dosed with 5 μm BAMs **1‐3** over the control (PBS) (Fig. 6A,B). This result is consistent with the hypothesis that the machinery activated in rodent models leading to dose‐dependent increases in spine density by the BAM agents is also present and can be activated by these compounds in a human neuronal model.

We next sought to examine whether BAMs **1‐3** could elicit the same phenotypic increase in PSD95 puncta in human neurons as was previously observed in rat primary hippocampal neuronal culture 14. For this experiment, we dosed 3‐month‐old neurons derived from iPSCs with 0 or 5 μm BAMs **1‐3** for 24 h. Neurons were then fixed, and immunochemistry was performed to examine the relative density of PSD95‐positive puncta located along MAP2‐labeled segments of dendrites (Fig. 6C,D). Here, a 1.5‐ to 2‐fold increase in the density of PSD95 puncta was observed for neurons treated with BAMs **1‐3** compared to the control neurons (incubated with PBS alone). These data support that BAMs **1‐3** exhibit similar phenotypic activity for promoting dendritic spines as had previously been observed in rat primary hippocampal neurons 14. These data also further support the utility of iPSC‐derived neurons to evaluate both the biochemical and phenotypic activities of promoters of dendritic spine formation in a non‐immortalized human cell model.

## Conclusion

We have examined the capability of BAMs to promote the formation of PSD95‐positive puncta in three different human cellular models: (a) undifferentiated, (b) differentiated SH‐SY5Y neuroblastoma cells and (c) neurons derived from human iPSCs. We found that both of the undifferentiated and differentiated neuroblastoma cell preparations were not suitable to examine both the biochemical and phenotypic activities of the BAM compounds as promoters of dendritic spine formation. In neurons derived from differentiating iPSCs, however, we were indeed able to observe both significant increases in RasGRF1 expression and phenotypic increases in dendritic spine density (as estimated by the density of PSD95‐positive puncta along MAP2‐labeled dendrites) when cells were exposed to the BAM compounds. These results demonstrate that iPSC‐derived neurons can serve as an accessible human model for examining the activity of compounds that affect dendritic spine dynamics. Additionally, these results further support that the analogous cellular machinery activated by BAM compounds in rodent hippocampal neurons may also be activated in human neurons, suggesting the possible utility of BAM agents to improve memory and learning through promoting dendritic spine formation for the treatment of human NDDs and other memory impairment disorders.

## Conflict of interest

The authors declare no conflict of interest.

## Author contributions

JLC and JY conceived the research. JLC and JY designed and JLC performed the experiments. JLC, KRB, and JY analyzed and interpreted the data. JLC, KRB, and JY wrote the manuscript.

## Data Availability

All data generated or analyzed during this study are included in this manuscript.
